# The Photodetectors Based on Lateral Monolayer MoS_2_/WS_2_ Heterojunctions

**DOI:** 10.1186/s11671-021-03581-4

**Published:** 2021-07-31

**Authors:** Caihong Li, Juntong Zhu, Wen Du, Yixuan Huang, Hao Xu, Zhengang Zhai, Guifu Zou

**Affiliations:** 1 Institute of Fundamental and Frontier Sciences, University of Electronic Science and Technology of China, Chengdu, 610054 People’s Republic of China; 2 the College of Energy, Soochow Institute for Energy and Materials Innovations, and Key Laboratory of Advanced Carbon Materials and Wearable Energy Technologies of Jiangsu Province, Soochow University, Suzhou, 215006 People’s Republic of China; 3 School of Physics, University of Electronic Science and Technology of China, Chengdu, 610054 People’s Republic of China; 4 the State Key Laboratory of Electronic Thin Films and Integrated Devices, University of Electronic Science and Technology of China, Chengdu, 610054 People’s Republic of China; 5 the 36th Research Institute of China Electronics Technology Group Corporation, Jiaxing, 314033 People’s Republic of China

**Keywords:** Lateral monolayer heterostructure, MoS_2_/WS_2_ heterojunction, Photodetector, Sharp interface

## Abstract

Monolayer transition metal dichalcogenides (TMDs) show promising potential for next-generation optoelectronics due to excellent light capturing and photodetection capabilities. Photodetectors, as important components of sensing, imaging and communication systems, are able to perceive and convert optical signals to electrical signals. Herein, the large-area and high-quality lateral monolayer MoS_2_/WS_2_ heterojunctions were synthesized via the one-step liquid-phase chemical vapor deposition approach. Systematic characterization measurements have verified good uniformity and sharp interfaces of the channel materials. As a result, the photodetectors enhanced by the photogating effect can deliver competitive performance, including responsivity of ~ 567.6 A/W and detectivity of ~ 7.17 × 10^11^ Jones. In addition, the 1/f noise obtained from the current power spectrum is not conductive to the development of photodetectors, which is considered as originating from charge carrier trapping/detrapping. Therefore, this work may contribute to efficient optoelectronic devices based on lateral monolayer TMD heterostructures.

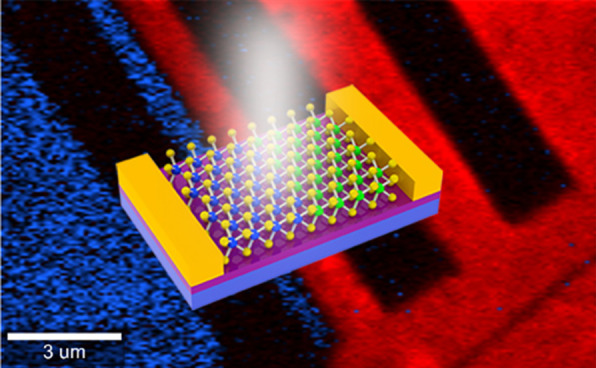

## Introduction

Considering the almost half-a-trillion-dollar semiconductor-chip market, two-dimensional (2D) materials are currently one of the most feasible and promising candidates for extending Moore’s law [1–5]. As a representative member of the 2D family, transition metal dichalcogenides (TMDs) have been intensively studied due to their distinctive optoelectronic properties and potential applications [6–12] in photodetection and light-emitting devices [13, 14]. Notably, the tunable bandgap, high carrier mobility, high optical absorption and atomically thin thickness, making TMDs appropriate channel materials for photodetectors, play a crucial role in optoelectronic or electronic devices [15, 16]. Although crystal defects in TMDs giving rise to the carrier trapping effect can result in high photosensitivity, they can unavoidably lead to slow response speed yet [17]. In addition, some researchers propose the plasmonic enhancement to boost the limited light utilization of 2D materials [18–20]. Combining respective superiorities and showing unique electronic transport at the junction, TMDs heterostructures either lateral stitching or vertical stacking are presented [21]. Such heterostructures can tailor intrinsic electronic properties and improve the optical absorption [22], showing emerging and designable features [13, 23]. For example, the built-in electrical field [24] or energy level difference [25] induced by TMD heterostructures should accelerate photocarrier separation [26], suppress photocarrier recombination [17, 27] and lower dark current [28] as well, which is beneficial for achieving high-performance photodetection. Besides, Wang’s group [29] has certified suppressed electron–hole (e–h) recombination in lateral heterostructures. As previously reported, the lateral heterostructures showed higher carrier mobility [30] whereas the vertical heterostructures usually increased the photoactive area [27] and/or enhanced current drive per area [31]. Moreover, the in-plane interfaces of lateral heterostructures showed stronger emission intensity than both sides [14]. However, the suppressed photoluminescence (PL) emission could be observed in the vertical hetero-interface because of the reduced direct radiative recombination [32]. Additionally, both lateral and vertical TMDs heterostructures make it possible to create new excitonic transitions [14].

In terms of crystal lattice quality, MoX_2_/WX_2_ (X = S, Se or Te) lateral heterojunctions could induce structural defects scarcely due to their similar honeycomb-like [33, 34] configuration and lattice parameters [34]. In addition, this kind of heterojunction can form type-II band alignment generally, which is desirable for high-efficiency photodetection [32, 34, 35]. According to the former work, lateral monolayer MoS_2_/WS_2_ heterojunction preferred to exhibit type-II band alignment with the valence band maximum (VBM) localized at WS_2_ and the conduction band minimum (CBM) at MoS_2_ [32, 34]. For instance, Wu’s group have further reported that the VBM and CBM of MoS_2_ are 0.39 eV and 0.35 eV lower than that of WS_2_, respectively [34]. Furthermore, the band offset between MoS_2_ and WS_2_ determining the band alignment could be estimated via their different d-orbital positions of Mo and W [34]. Vertical heterostructures can be prepared by mechanical transfer and stack, whereas lateral ones can be only achieved by growth methods [14]. Furthermore, vertical heterostructures, as previously reported, cannot be precise control and it is easily contaminated at the interfaces between layers [33]. Luckily, the lateral heterostructures can be synthesized by one-step method to reduce contaminations [28]. Nowadays the growth of large-area and high-quality lateral monolayer TMDs heterostructures has remained a great challenge [36]. Hence, high-quality and large-area lateral TMDs heterojunctions are significant and desired for the development of high-performance photodetectors.

Here, the lateral monolayer MoS_2_/WS_2_ heterojunctions with sharp interfaces and good uniformity via one-step liquid-phase CVD method are prepared and photodetectors are fabricated based on these heterostructures. The presented photodetectors can deliver high responsivity and detectivity of 567.6 A/W and 7.17 × 10^11^ Jones, respectively. This work demonstrates lateral monolayer MoS_2_/WS_2_ heterojunctions can serve as qualified candidates for next-generation optoelectronic applications.

## Methods

### Heterostructure Synthesis

0.05 g sodium tungstate, 0.5 g ammonium molybdate and 0.12 g NaOH (or KOH) particles were dissolved in 10 mL of deionized (DI) water to obtain precursor solution. The growth substrates (sapphire) were treated by piranha solution to improve the surface hydrophilicity, and then the precursor solution was evenly spin-coated onto clean sapphire substrates. After that, the precursor covered sapphire and sulfur were placed on the heating center and upstream of a quartz tube, respectively. The heating center was ramped up to 700 °C in 40 min and maintained for 10 min to grow MoS_2_-OH bilayers (i.e. MoS_2_ monolayer and a single layer of OH^−^ ions attached). Finally, the carrier gas was changed from Ar to Ar/H_2_ (5% H_2_), and the heating center heated to 780 °C within 10 min and kept for 10 min to allow WS_2_ to grow along the edges of MoS_2_–OH bilayers, forming MoS_2_/WS_2_ lateral heterostructures. The more details of the heterostructure synthesis refer to previous work [30].

### Transfer Process

We used the polystyrene (PS)-assisted method to transfer WS_2_/MoS_2_ lateral heterostructures from sapphire to SiO_2_/Si substrates. The PS solution (9 g of PS was dissolved in 100 mL of toluene) is first spin-coated on the heterostructures with 3500 rpm for 60 s, then the sample is baked at 90 °C for 10 min to eliminate toluene. After that, the WS_2_/MoS_2_–PS film is obtained by a water droplet, and the floating WS_2_/MoS_2_–PS film is then dredged up with a clean SiO_2_/Si substrate. The WS_2_/MoS_2_–PS-SiO_2_/Si sample is baked at 80 °C for 1 h and then at 150 °C for 30 min to spread the polymer to eliminate possible wrinkles. Finally, the PS film is removed by rinsing with toluene several times to get WS_2_/MoS_2_-SiO_2_/Si samples.

### Device Fabrication

The standard electron beam lithography (EBL) was used to define the markers and electrode patterns on the as-grown lateral monolayer MoS_2_/WS_2_ heterojunctions. The Ti/Au electrodes (10 nm/100 nm) were evaporated on the channel and lifted off in acetone. The device was thermal annealed at 400 °C for 2 h in vacuum and cooled down to room temperature rapidly.

### Material Characterization

The optical images were captured with OLYMPUS microscope (LV100ND). The Raman, PL and AFM mapping images were measured with a Raman-AFM confocal spectrometer (Witec, alpha300 RA) with a laser of 532 nm.

### Device Characterization

The optoelectronic properties of the photodetectors were measured with the SemiProbe probe station and a semiconductor parameter analyzer (Keithley 4200) and Platform Design Automation (PDA, FS- Pro). Different wavelength lasers as the light sources were used to measure the photoresponse of the photodetectors. Different laser densities were determined with an irradiatometer.

## Results and Discussion

**Fig. 1:**
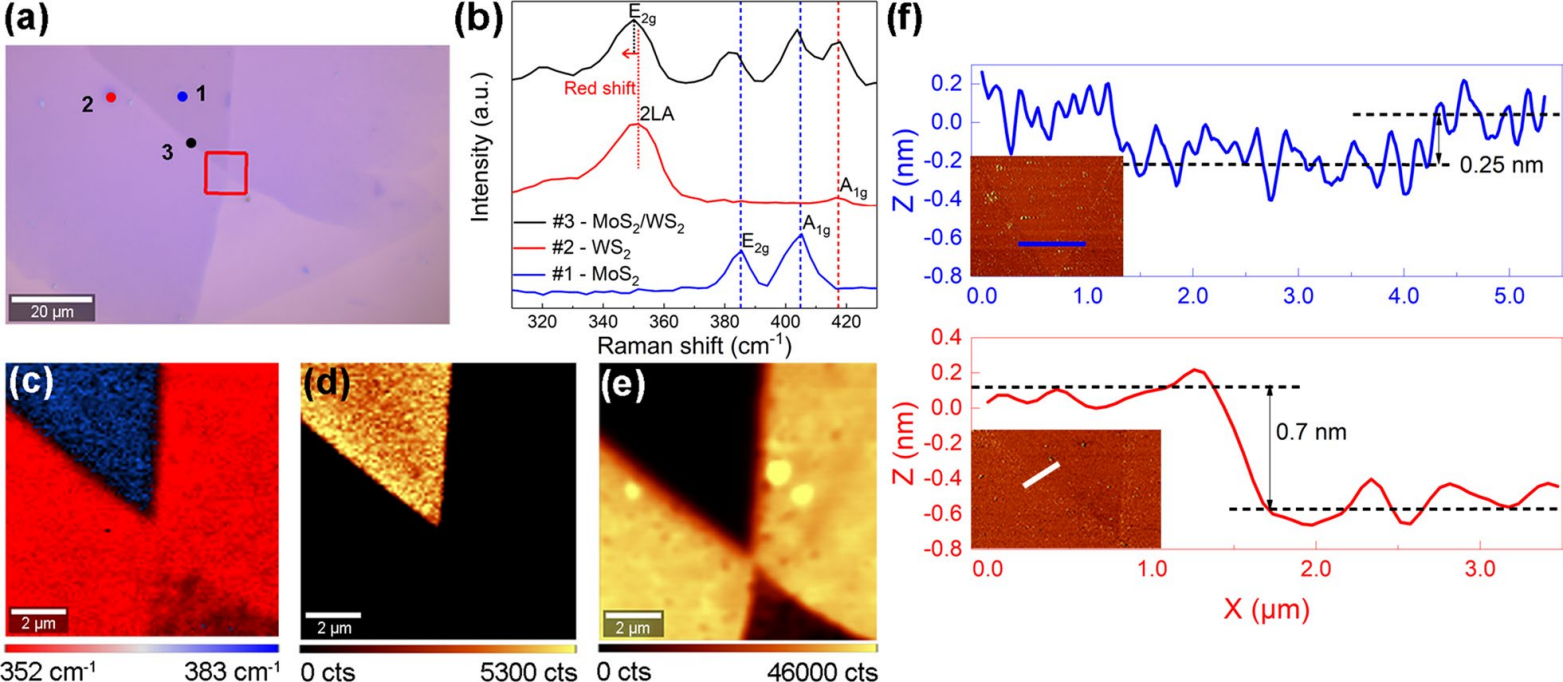
Material characterization results of the as-grown lateral monolayer MoS_2_/WS_2_ heterostructure. (**a)** The optical image of the lateral monolayer MoS_2_/WS_2_ heterojunction. (**b)** The Raman spectrum obtained from the site marked with 1, 2 and 3 in (**a**), respectively. The Raman mapping image (**c**), PL mapping images of MoS_2_ region (**d**) and WS_2_ region (**e**) from the red framed area in (**a**). The corresponding false-color bar is inserted at the bottom of (**c**)–(**e**). (**f**) The corresponding cross-sectional height profile of the blue (between WS_2_ and MoS_2_) and white (between WS_2_ and substrate) lines marked in AFM morphology image

Figure 1a shows the optical image of the CVD-grown lateral monolayer heterostructure, illustrated by the optical contrast. The corresponding Raman spectra obtained from the different positions marked 1 and 2 in Fig. 1a confirm the configuration of the inner MoS_2_ (385.5 cm^−1^ and 405.3 cm^−1^) and outer WS_2_ (351.5 cm^−1^ and 416.5 cm^−1^) in Fig. 1b [30]. High crystal quality of MoS_2_ and WS_2_ are implied because no oxidation peak observed in the corresponding Raman spectra [37]. Especially, the eigen-peaks of MoS_2_ and WS_2_ both were observed in the stitched interface marked 3 in Fig. 1a, indicating two materials form at the interface. In addition, the frequency difference between the E_2g_ mode and A_1g_ mode of MoS_2_ is 19.8 cm^−1^, suggesting monolayer one [30, 38, 39]. When considering WS_2_, the peak intensity ratio of longitudinal acoustic mode (2LA) [40] at 352 cm^−1^ to A_1g_ mode, i.e. I_2LA_/I_A1g_, is more accurate to verify the thickness than frequency difference [14]. The ratio was estimated to be ~ 2, in agreement with monolayer WS_2_ measured by 532 nm laser [14]. The distinct red shift of E_2g_ mode (in-plane vibration) can be observed, resulted from alloying effect [41] in the lateral heterojunctions. Notably, this similar behavior were also observed in the vertical heterojunctions, caused by dielectric screening and interlayer coupling [42]. Furthermore, the Raman mapping result in Fig. 1c with the blue region of MoS_2_ and the red region of WS_2_ indicates the seamless high-quality in-plane heterostructure [13, 43]. Figure 1d, e also demonstrate the configuration with MoS_2_ inside and WS_2_ outside by PL mapping, respectively [13]. Several points showing enhanced PL intensities in WS_2_ region may be explained as carrier inhomogeneity caused by impurities or vacancies [14]. In addition, the stronger PL emissions at the interface than the MoS_2_ region could be interpreted as the inhomogeneous distribution of carriers or higher photoinduced carrier recombination rate at the edges [14]. Both Raman and PL mapping suggest a sharp and well-stitched interface between MoS_2_ and WS_2_ [14, 44]. The thickness and surface morphology were measured by atomic force microscope (AFM) with trapping-mode. Note that few grain boundaries resulting in charge carrier scatting [45] are observed in material inside but edges indicating better electrical transport performance as shown in Fig. 1f [14, 46]. The thickness of WS_2_ outside is ~ 0.7 nm (bottom) consistent with CVD-grown WS_2_ monolayer reported previously [47], and the height difference between WS_2_ and MoS_2_ is about 0.25 nm (top), implying monolayered MoS_2_ [47]. Overall, the above material characterization results can demonstrate the lateral monolayer MoS_2_/WS_2_ heterojunction with the sharp interface.

**Fig. 2:**
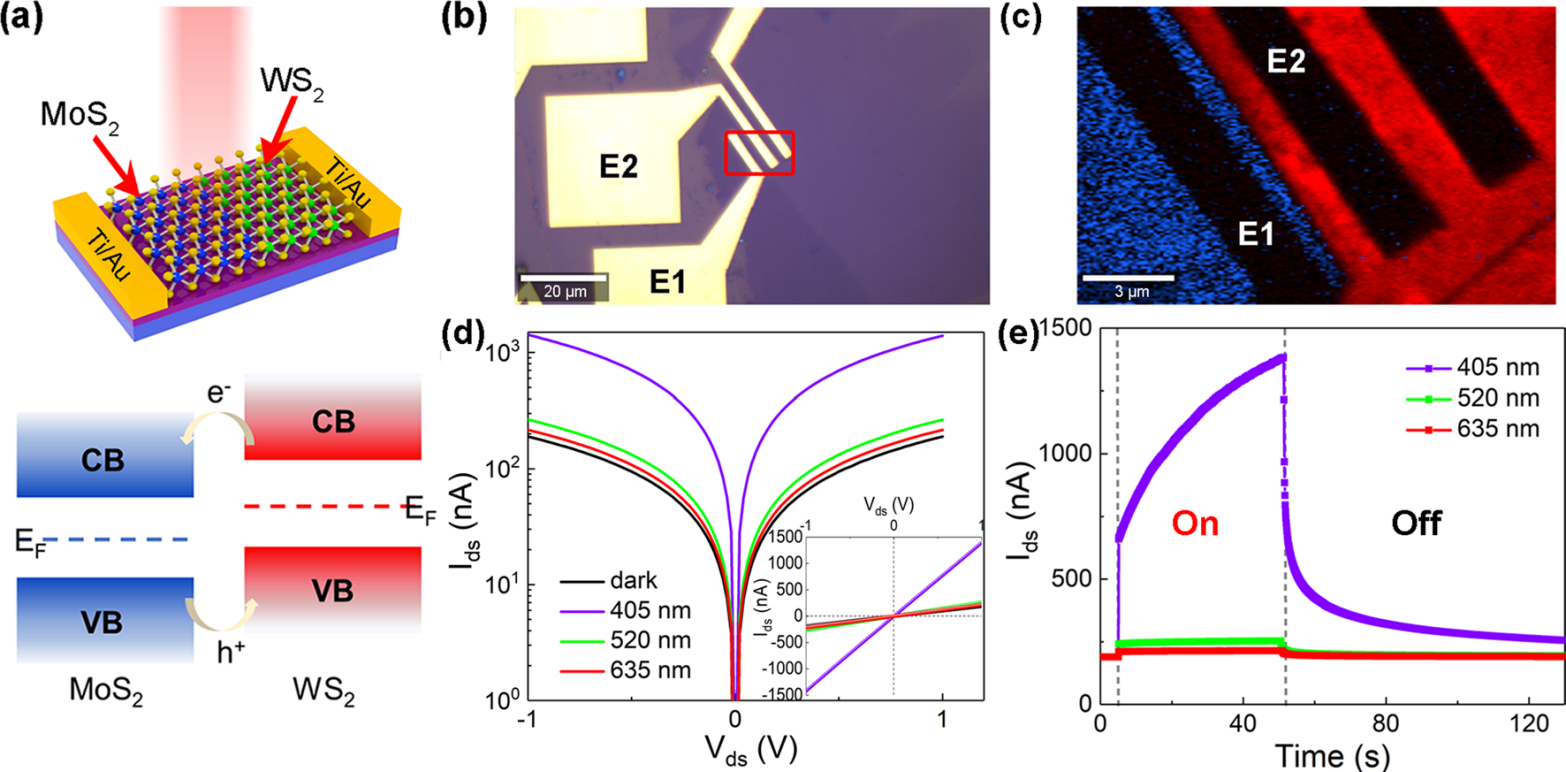
Optoelectronic characteristics of the photodetector. (**a)** The schematic diagram and proposed band alignment of the photodetector. The optical image (**b**) and corresponding combined Raman mapping (**c**) of the photodetector. E1 and E2 represent the source and drain electrodes of the measured device. The semi-logarithmic (**d**) and linear (inset of (**d)**) *I*–*V* characteristics and the photoswitching characteristics (**e**) of the photodetector

Photodetectors were fabricated using an EBL system based on the lateral MoS_2_/WS_2_ heterojunction. Figure 2a exhibits the schematic diagram (top) of the lateral heterojunction device and corresponding type-II band alignment (bottom). Accordingly, electrons and holes are transferred and confined in MoS_2_ and WS_2_ region through the interface, respectively, achieving the photoelectric conversion [13, 21, 24, 48]. We attribute this to the photogating effect, such as a special case of photoconductive effect [49]. The photogating effect can work as a local photogate modulating channel conductance [50]. The optical image of the device with the effective device area of ~ 40 μm^2^ is described in Fig. 2b with E1 and E2 electrodes as the source and drain electrodes. In order to figure out the heterojunction configuration, combined Raman mapping was carried out (Fig. 2c), indicating the channel materials of lateral MoS_2_/WS_2_ heterojunction between the measured source and drain electrodes (E1 and E2) [28]. The blue, red and dark sections are MoS_2_, WS_2_ and metal electrodes, respectively. Figure 2d shows the semi-logarithmic output characteristic curves of the lateral heterojunction under visible light with 405 nm, 520 nm and 635 nm, respectively. The inset in Fig. 2d reveals a linear *I-V* relationship between the channel and the electrodes [51–56]. The linear *I*–*V* character is conducive to achieving high responsivity but poor sensitivity of photodetectors due to a high dark current [57]. Additionally, the *I*_ph_ (i.e. *I*_light_ – *I*_dark_) of the photodetector increases to 12.5 times of that before thermal annealing, which maybe ascribe to decreased contact resistance [46, 58], removal of defects [59] and improved electrical conductivities [60]. Figure 2e depicts the photoswitching characteristics excited by the above wavelengths. The transient current rises rapidly when the light is on and drops as soon as the light is off, implying this photodetector can serve as a prompt light-activated switch [61].

**Fig. 3:**
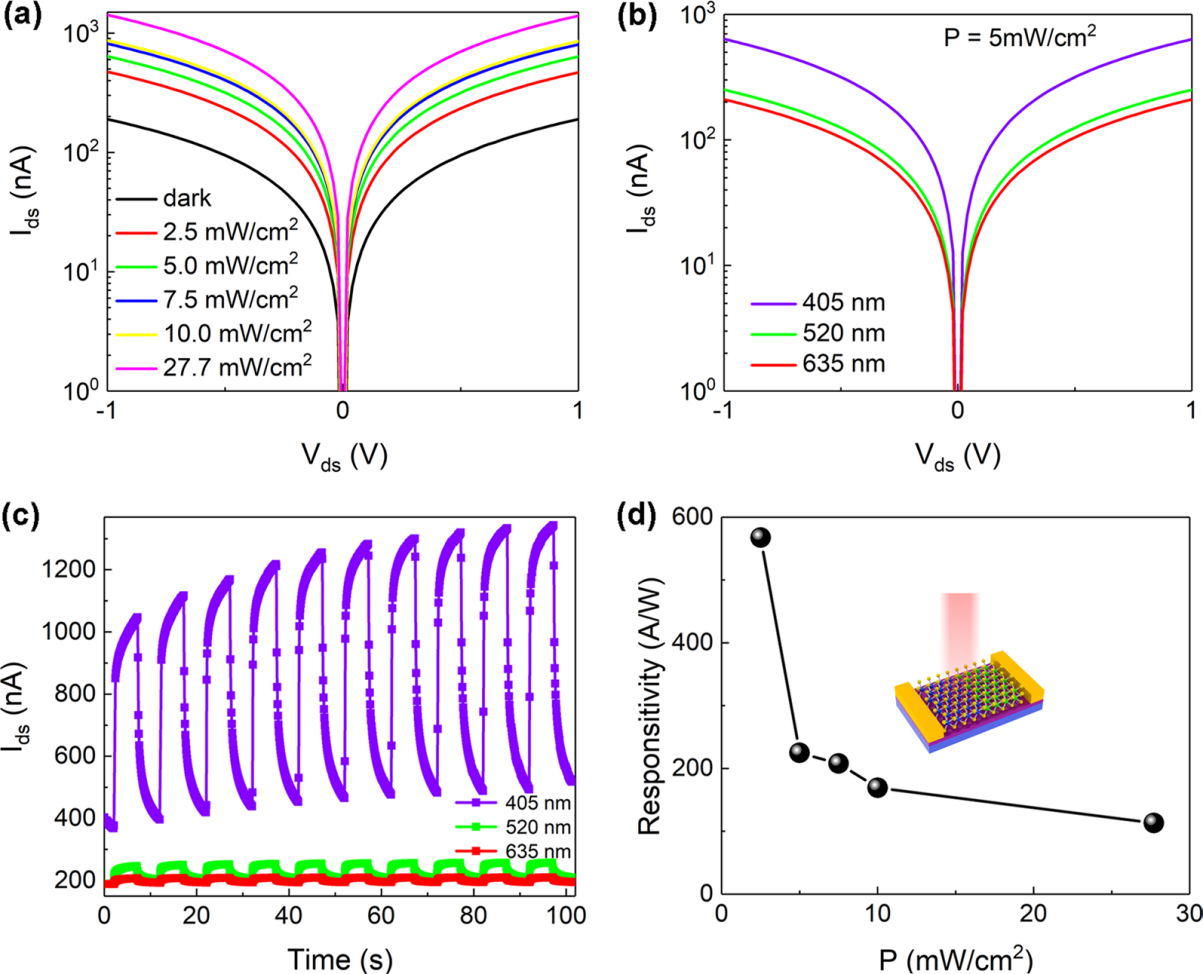
Photoresponse behavior of the photodetector. The *I*–*V* characteristics under different 405 nm laser power densities (**a**) and under different incident wavelengths of 5 mW/cm^2^ (**b**). (**c)** The time-resolved photoresponse excited by the periodic on/off switching of incident light. (**d)** The extracted *R* (black sphere) as a function of laser power densities. The applied voltage for (**c**–**d**) is 1 V

The semi-logarithmic output characteristics with the same wavelength but varied laser power densities are depicted in Fig. 3a. As expected, photocurrent is enlarged as the laser power densities increase due to more induced photogenerated carriers [62]. Figure 3b shows the *I*–*V* curves with the same laser power density but different incident wavelengths (i.e. different light absorption amount and optical excitation energy). Although the shorter wavelength possesses fewer photons compared to the longer wavelength at the same laser power density. In this instance, the measured transient current increases with the decreases of the irradiation wavelength. This may be caused by the reduced optical absorption at the longer wavelength [63, 64]. Figure 3c describes the transient current under periodic laser illumination of 10 s, indicating a stable reproducible photoresponse [61]. For most low dimensional photodetector dominated by photogating effect, limited response speed and high responsivity can be obtained due to the prolonged excess carrier lifetime [50, 65]. The rise/fall time is defined as the time required for the photocurrent to rise/fall from 10%/90% of the stable value to 90%/10% [66, 67]. The relatively long rise/fall time should be caused by slow carrier recombination, originated from laser illumination exciting many defective states [68]. Therefore, the response time including rise time and fall time was sacrificed by photogating effect because of the long-lived charge trapping processes [57]. Some researchers have proposed that the high-quality channel material which can offer a smooth and short path for carrier transfer and optimal device structure can improve the response speed [69, 70]. Indeed, the figures of merit of the photosensitive devices are mainly responsibility (*R*) and detectivity (D*). *R* is calculated by the relations of

1 $$R = {{\mathop I\nolimits_{ph} } \mathord{\left/ {\vphantom {{\mathop I\nolimits_{ph} } {(P \cdot S)}}} \right. \kern-\nulldelimiterspace} {(P \cdot S)}}$$

where *P* and *S* are laser power density and effective device area, respectively [62, 71, 72]. Figure 3d shows the corresponding values of *R* of the photodetector under different laser power densities. The champion *R* reaches up to ~ 567.6 A/W delivering the competitive performance parameter. The high *R* is attributed to the suppressed photocarrier recombination in the heterostructure together with electron trapping in the MoS_2_ region presumably [22]. The decreased *R* as the laser power density increased reveals the photogating effect in the photodetector further [73].

**Fig. 4:**
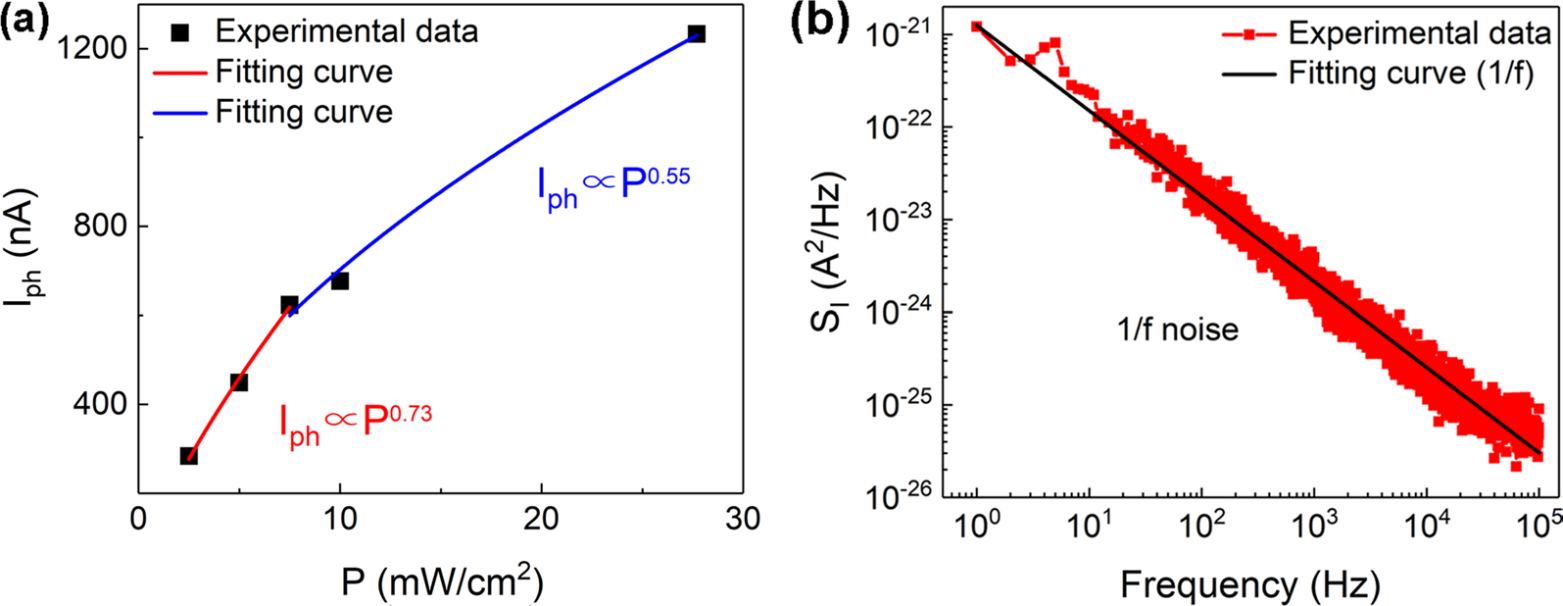
**(a)** The plot of *I*_ph_ versus laser power densities. (**b)** The current power spectrum (*S*_*I*_) under different frequencies. The applied voltage for (**a–b**) is 1 V

Moreover, photocurrent and laser power density follow the power-law equation:

2 $$\mathop I\nolimits_{ph} = A\mathop P\nolimits^{\alpha }$$

where *A* is a constant and 0 < *α* < 1. The value of *α*, obtained by fitting the curve of *I*_ph_ versus *P* in Fig. 4a, is related to the process of carrier capture, recombination and transfer [74, 75]. The sublinear relation between *I*_ph_ and *P* suggests the presence of the photogating effect in the device further [65]. The higher value of α (such as ~ 0.73) can be obtained when the lower power densities are applied due to reduced photocarrier recombination and the interactions between carriers [75, 76]. In contrast, higher power densities can result in a degraded α value of ~ 0.55 because of stronger recombination losses and more trap states [77]. The precondition of the calculated D* via the equation

3 $$\mathop D\nolimits^{*} = R\mathop {({S \mathord{\left/ {\vphantom {S {2e\mathop I\nolimits_{{{\text{dark}}}} }}} \right. \kern-\nulldelimiterspace} {2e\mathop I\nolimits_{{{\text{dark}}}} }})}\nolimits^{{{1 \mathord{\left/ {\vphantom {1 2}} \right. \kern-\nulldelimiterspace} 2}}}$$

is that the photodetectors are limited by shot noise as the main noise source [49, 66, 78]. In order to further evaluate D* more accurately, the noise current obtained in Fig. 4b is measured under different frequencies [74]. Figure 4b shows the typical 1/f noise [79] in our photodetectors, which is significant impediment to semiconductor industry from new materials. This kind of noise is mainly resulted from the charged impurities and trapping sites in the conductive channel [57, 80]. A higher material quality and small structural defect density are desired for reducing the 1/f noise [81]. According to the formula of

4 $$\mathop D\nolimits^{*} = R{{\mathop {(S\Delta f)}\nolimits^{{{1 \mathord{\left/ {\vphantom {1 2}} \right. \kern-\nulldelimiterspace} 2}}} } \mathord{\left/ {\vphantom {{\mathop {(S\Delta f)}\nolimits^{{{1 \mathord{\left/ {\vphantom {1 2}} \right. \kern-\nulldelimiterspace} 2}}} } {\mathop I\nolimits_{{{\text{noise}}}} }}} \right. \kern-\nulldelimiterspace} {\mathop I\nolimits_{{{\text{noise}}}} }}$$

where Δ*f* and *I*_noise_ are measurement bandwidth and noise current [79], the detectivity of the photodetector is about 7.17 × 10^11^ Jones. Table 1 has compared some selected representative photodetectors with corresponding photoresponse performance based on 2D materials. The relatively high *R* and D* of our photodetectors show great potential in optoelectronic devices.

**Table 1:** Some photoresponse performance of selected representative photodetectors based on 2D materials

Materials	Wavelength (nm)	Responsivity (A/W)	Detectivity (Jones)	References
monolayer WSe_2_	650	3.5 × 10^5^	10^14^	[82]
MoS_2_-on-Au	1310	0.68	1.89 × 10^12^	[83]
WSe_2_/SnS_2_ heterostructure	550	244	1.29 × 10^13^	[84]
MoS_2_/MoSe_2_ heterojunction	610	1.3	2.6 × 10^11^	[85]
WSe_2_/WS_2_ heterojunction	532	300	7 × 10^2^	[86]
SnSe_2_/MoS_2_ heterostructure	500	9.1 × 10^3^	9.3 × 10^10^	[87]
WSe_2_/BP/MoS_2_	532	6.32	1.25 × 10^11^	[88]
CdS_x_Se_(1–x)_	450	703	3.41 × 10^10^	[89]
SnS_2x_Se_2(1–x)_	633	6 × 10^3^	8.2 × 10^12^	[90]
WS_2_/graphene nanosheets	480	1.15	2.06 × 10^9^	[91]
SnS_2_ nanoflakes	400	354.4	2.0 × 10^10^	[92]
SnSe_2_ flakes	530	1.1 × 10^3^	1.01 × 10^10^	[93]
Ca_2_Nb_3_O_10_	280	14.94	8.7 × 10^13^	[72]
Sr_2_Nb_3_O_10_	270	1214	1.4 × 10^14^	[71]
Graphene/WS_2_ heterostructure	400–700	10^6^	3.8 × 10^11^	[94]
P-MoS_2_/N-MoS_2_	635	7 × 10^4^	3.5 × 10^14^	[95]
MoS_2_/α-MoO_3-x_	405	1.9 × 10^5^	9.8 × 10^16^	[96]
MoS_2_/WS_2_ heterojunction	405	567.6	7.17 × 10^11^	This work

## Conclusions

In summary, a high-performance photodetector was developed based on the lateral monolayer MoS_2_/WS_2_ heterojunction. The size of the channel materials grown by the one-step liquid-phase CVD method reaches up to millimeter scale. Moreover, the high-quality channel materials with good uniformity and sharp interface were examined by systematic material characterizations and subsequent device measurements. Particularly, high responsivity of 567.6 A/W and detectivity of ~ 10^11^ Jones are achieved for the photodetectors attributing to the photogating effect. The performance of the proposed lateral MoS_2_/WS_2_ heterojunction photodetectors is better than or comparable to the reported work [24, 62, 76, 78, 86, 97, 98]. In addition, we suppose the undesired 1/f noise arising from the trapping/detrapping of charge carriers maybe further reduced by high-quality and defect-less channel material. The facile one-step liquid-phase CVD growth and excellent optoelectronic performance of the photodetectors can motivate further research regarding optoelectronic devices based on lateral heterostructures.

## Data Availability

The datasets supporting the conclusions of this article are included in the article.
